# Fun and a meaningful routine: the experience of physical activity in people with dementia

**DOI:** 10.1186/s12877-022-03149-6

**Published:** 2022-06-10

**Authors:** Elisabeth Wiken Telenius, Gro Gujord Tangen, Siren Eriksen, Anne Marie Mork Rokstad

**Affiliations:** 1 The Norwegian National Centre for Ageing and Health, Vestfold Hospital Trust, PO Box 2136, 3103 Tønsberg, Norway; 2 Faculty of Health Studies, VID Specialized University, Oslo, Norway; 3 Department of Geriatric Medicine, Oslo University Hospital, Oslo, Norway; 4 Lovisenberg Diaconal University College, Oslo, Norway; 5 Faculty of Health Sciences and Social Care, Molde University College, Molde, Norway

**Keywords:** Dementia, Physical activity, Exercise, Interview, Experience, Need

## Abstract

**Background:**

Physical activity is important to health and wellbeing. People with dementia are less physically active than their cognitively healthy counterparts. Reasons for this are multifaceted, and are thought to be social, psychological, and physiological. People with dementia often use services such as home care, day care centres and nursing home, and according to the stage of disease they are less or more dependent on other people to take part in activities. To develop appropriate services to this patient group, their needs and preferences regarding physical activity must be recognized. The aim of the study was therefore to provide insight into experiences with physical activity in people with dementia.

**Methods:**

The current study is part of a larger research project on needs in people with dementia. The main project included qualitative semi-structured interviews with 35 persons with dementia. 27 of the participants talked about their experience with physical activity. In the current study, the relevant findings on this theme were analysed separately. A phenomenological hermeneutic research design was applied.

**Results:**

The analysis revealed three main categories regarding experiences with physical activity. To be physically active provided *positive experiences* such as feelings of mastering and post-exercise euphoria. To be physically active was *meaningful*. The daily walk was an important routine to many, and it gave meaningful content to the day. Keeping up with activities confirmed identity. Lastly, to be active was perceived as *challenging*. Participants described different barriers to being physically active such as a decline of physical function, lack of motivation and being dependent on others to go out.

**Conclusions:**

Many of the participants expressed that being physically active was important to them. It is essential that informal and formal carers are aware of the role physical activity plays in the lives of many people with dementia, so that appropriate measures can be taken to assure continued active living in order to preserve health and quality of life.

**Supplementary Information:**

The online version contains supplementary material available at 10.1186/s12877-022-03149-6.

## Background

The United Nations has proclaimed 2021–2030 as the decade of healthy ageing. The importance of staying active during ageing has been articulated by many and WHO has developed guidelines for physical activity specifically for older adults [1]. Physical activity has been defined as any voluntary movement produced by the skeletal muscles that result in increased energy expenditure, and exercise is described as a subcategory of physical activity which is planned, structured, and repetitive, with the intent of improving or maintaining one or more patterns of physical fitness or function [2]. In old age, physical activity can prevent decline in physical and cognitive function and improve mastery [3–5]. Even though the vast benefits of exercise and physical activity are well documented and established, many older people fail to reach the level of physical activity as recommended by WHO [6]. People of all ages experience barriers to physical activity, however older individuals report stronger and more barriers than middle-aged people [7]. Various barriers to physical activity include fear and negative experiences, lack of company and an unsuitable environment, disabilities, poor balance, fear of injury and depressive symptoms [8–10].

Recent studies demonstrate that physical activity can be particularly important for people with dementia [11–13]. Nevertheless, it has been established that they live more inactive lives than age matched cognitively intact counterparts [14, 15]. Although to some extent equivocal, the literature suggest that physical exercise can influence balance, gait function, cognition and behavioural and psychological symptoms experienced by this patient group. Outdoor activities have been found to contribute to well-being and feelings of self-worth among people with dementia [16]. Also, the social aspect of physical activity is important to many. Social relationships play a significant role in maintenance of self [17, 18].

Besides experiencing the barriers to physical activity as described in the general older population, people with dementia face additional barriers. Examples are memory problems and confusion, dependence on others and lack of access to dementia-specific exercise programmes [19–22]. Also, apathy and lack of initiative are common symptoms in early dementia [23]. Gait and balance impairments increase during the course of dementia, which can be a barrier to activity, but at the same time makes physical activity even more important for this group [24]. Since physical inactivity is one of the leading risk factors for frailty and mortality, it is important to lessen barriers and facilitate participation in physical activities for this group of people [25, 26].

A few studies have reported on the experience of people with dementia on the subject of physical activity and exercise. McDuff and Phinney (2015) interviewed 12 participants with dementia and found that physical activity remains important although the persons are confronted with barriers such as physical discomfort, lack of enthusiasm and memory loss [27]. They found that the participants gave the following reasons to be active: improved well-being, was social, gave opportunity to be outdoors and provided purpose and structure to the day. This is supported by Cedervall (2015) [28]. Malthouse and Fox (2014) interviewed people with dementia together with spouses about barriers and facilitators to physical activity [29]. They found that the experience of physical activity could be influenced by factors at individual level, social relations, and relationship between person with dementia and carer. These interview studies showed that it is important to people with dementia that physical activity programmes are tailored to the needs and interests of the participants [20, 29, 30].

Person-centred care is a world-wide care approach of choice to develop high quality dementia care [31]. In person-centred care the person’s preferences and life history are central in the development and implementation of care and services [32]. People with dementia are often capable of communicating their views and preferences about what is important to them [33, 34] and it is ethically and morally important for service providers and informal carers to request and consider those views. The present study investigated needs in persons with dementia, and many of the participants talked about physical activity and exercise in this context. The subjective experience of physical activity for people with dementia has not received much attention in the research literature. Hence, the aim of this study was to explore the experiences of people with dementia regarding physical activity and exercise.

## Method

### Research design

The current study is part of a larger research project named “The needs of people with dementia”. The project aimed to explore the needs from different angels expressed by people with dementia themselves, their family caregivers and health care professionals. Semi-structured interviews with open-ended questions in line with Kvale and Brinkmann (2009) were carried out to explore the experienced needs of people living with dementia [35]. Physical exercise and other forms of physical activity were described as significant needs by many of the participants as the topic came up in most interviews. The current article reports on an in-depth analysis of data from the sample of participants who described physical activities as an important need.

### Recruitment

Out of the sample in the main project of 35 persons, 27 spoke of their experience with physical activity and physical exercise; 16 women and 11 men aged between 59 and 92 years. This means that 8 interviews were excluded from the main sample. The participants were recruited through health care professionals who provide services for people with dementia in primary and specialised health care. Each of them was diagnosed with dementia in primary or specialised care. The type of dementia disorder and severity were not recorded as this information was outside the scope of the study, however, the reported need for services can to a certain degree advise us on the severity of dementia condition. In most instances (*n* = 24) the first contact was made over telephone with family carer who had received information about the project from health care professionals who delivered services to people with dementia. Additional information was provided and appointment for interview was made.

### Pre-understanding

First and second authors (EWT and GGT) are physiotherapists and concerned with research on physical exercise and physical function in dementia. The two co-authors (SE and AMMR) are both registered nurses with extensive experience within the research field of dementia care.

### Ethical approval and consent to participate

Before commencing the interview, the persons with dementia were given oral and written information about the project, as well as the opportunity to consent or decline participation. The participants were also distinctly informed about their right to terminate the interview at any time. All participants gave their written informed consent. They were considered competent to consent by contact person, next of kin and the interviewer. The transcribed interviews were de-identified and all names in this article are fictional. All methods were carried out in accordance with relevant guidelines and regulations. The study is approved by the NSD, Norwegian Centre for Research Data (project number 51712). The project was presented for the Regional Committee for Medical and Health Research Ethics. They concluded that it was not subject to approval in accordance with the Health Research Act (Reference 2016/1868/REK midt). Even though all participants in this study were considered competent to consent, interviewing people with dementia requires sensitivity and awareness. The interviewer (EWT) is experienced in research on participants with dementia. Interviewing people with dementia contains different ethical considerations, and it can be challenging. Some might have difficulty finding words and some lose track of the conversation due to their memory loss. This requires the interviewer to be able to keep the conversation going at the pace of the person with dementia and to act respectfully when participants need to be directed back to the topic [36, 37].

### Data collection

The first author carried out all the interviews supported by a semi-structured interview guide (Additional file 1: Appendix A). All but two interviews were completed in the participants’ home. Eighteen were interviewed alone while nine of the participants preferred to have their partner or a family member present, mainly due to communication challenges. The interviews lasted between 20 and 90 min and were digitally audio recorded with an Olympus VN5500 dictaphone. Transcriptions were performed verbatim. Sound files and transcripts were anonymized and stored in a secure database.

### Analysis

Transcribed interviews were analysed according to qualitative content analysis inspired by Graneheim and Lundman (2004) [38]. The data were in part managed by using the software Nvivo 11.

The analysis of the interviews was completed in six stages. Stage 1: transcripts from all 35 interview were read several times in order to identify themes that were relevant to the aim of the study. Eight interviews were excluded because they did not address the topic of physical activity or physical exercise. Stage 2: The texts were divided into meaning units. Stage 3: The meaning units were consolidated into depictions close to the text. Stage 4: The meaning units were extracted and labelled with codes. Stage 5: Codes were grouped into sub-categories. Stage 6: The subcategories were gathered and abstracted as categories.

## Results

From the 27 participants, 12 received no services while five participants stayed in sheltered care or nursing home. The remaining ten participants received home care services and/ or attended day care regularly. The participant characteristics are presented with fictive names in Table 1.

**Table 1:** The participants

Name^*^	**Age group**	**Marital status**	**Interview setting**	**Services**
William	75–79	Married	Alone	No services
Sofie	85–89	Married	Alone	No services
Kari	70–74	Cohabiting	Next of kin present	No services
Mia	70–74	Cohabiting	Alone	No services
Jacob	70–74	Lives alone	Alone	No services
Ella	65–59	Married	Next of kin present	No services
Julie	75–79	Married	Alone	No services
John	65–69	Cohabiting	Next of kind present	No services
Olivia	55–59	Married	Alone	No services
Karl	70–74	Cohabiting	Next of kin present	No services
Arthur	75–79	Married	Alone	No services
Marie	65–69	Married	Alone	No services
Hugo	60–64	Married	Alone	Day care centre
Emma	85–89	Widowed	Alone	Home care
Linda	80–84	Married	Alone	Day care centre
Amelia	90–94	Widowed	Alone	Sheltered care
Lisa	85–89	Widowed	Alone	Day care centre
Kristian	60–64	Cohabiting	Next of kin present	Sheltered care
Erik	80–84	Married	Alone	Day care centre
Johanne	90–94	Married	Alone	Respite care
Hannah	85–89	Widowed	Alone	Home care
Greta	90–94	Widowed	Next of kin present	Nursing home
Thomas	80–84	Married	Alone	Respite care
Alice	85–89	Married	Alone	Nursing home
Victor	75–79	Lives alone	Next of kin present	Home care
Frank	70–74	Married	Next of kin present	Day care centre
Isabella	80–84	Widowed	Next of kin present	Nursing home

^*^Names are fictive

The main objective of this study was to explore the experience of people with dementia regarding physical activity and exercise. The analysis revealed three main categories in the material: (1) To be physically active provides positive experiences; (2) To be physically active is meaningful and (3) To be physically active is challenging.

### To be physically active provides positive experiences

The participants experienced that physical activity strengthened the sense of competence and provided positive feelings and pleasure. To some, being outdoors and experiencing nature were important part of this positive experience.

The participants talked about how physical activity and exercise gave them positive experiences in different ways. Several explained that they compared their performance on physical exercise to that of others. Finding out that their physical fitness was as good as, or better than, their friends’ or partner’s, gave satisfaction and a feeling of mastery. This confirmation that they still “measured up” and were able to participate on the same terms as their peers provided self-esteem. This was expressed by Mia who said that she was happy to see that she could still keep up with her husband when they went for a walk. She emphasized that he did not have to wait for her too much while she experienced to fall short in other activities. In line with this, several of the participants were proud of their physical accomplishments.

Several participants talked about their fondness for being physically active outdoors. Lisa was in her late eighties and lived alone in a house with a big garden. She enjoyed being self-sufficient in keeping the garden and spent a lot of time outdoors during all seasons. She said:

*“I like to work outside (…) from shovelling snow to mowing the lawn and stuff”*.

She explained that it gave her great pleasure to observe the lawn afterwards and enjoy the results from the hard labour. John believed that walking outdoors could prevent depression. He said that he went for walks every day with his partner whatever the weather and explained it like this:

*“You can’t get *stuck* inside the house and get depressed”.*

Many of the participants described their love for spending time at the cabin. Traditionally in Norway, life at the cabin includes outdoor activities such as hiking, skiing, swimming, wood chopping and fishing. Even though the time spent on these activities was less than earlier, the participants said that they still did these activities while at the cabin.

Many of the participants talked about how physical exercise gave positive feelings and pleasure. Jacob who was in his early seventies stell led a very active life and took great pride in being independent in doing grocery shopping by bike and going mountain hiking with his girlfriend. Sometimes he went “all in” while exercising, and he said:

*«It’s good to torment the body a little bit because it feels so damn good afterwards…”.*

This was supported by Hugo who described how he enjoyed the feeling of walking fast up the hills of his town, and how he challenged himself and noticed that his body was responding to the exercise. The social aspect of being active was also emphasised by several of the participants. Some talked about the importance of meeting other people while doing exercises, others did not mind – or preferred to do it alone. Hugo, who was in his sixties, said that he had been reluctant to visit the day care centre, however he quickly found his place there much thanks to the physical activities. Hugo described it like this:

*“When we take walks we walk hard and thoroughly enjoy ourselves. We laugh and tell jokes and have a great time”.*

The social aspect of exercise was also emphasised by Olivia and William who talked about how they appreciated the company of a friend when they were active outdoors.

### To be physically active is meaningful

The participants spoke of different ways that being active and exercising gave meaning to their day: Physical activity gave content to the day, reinforced their identity and was a way of controlling their life and situation.

The participants described how their daily walk was a routine that gave rhythm and content to the day. One of the participants explained that he enjoyed virtual bike trips every time he went to the day care centre. He used a stationary bike and “visited” locations around the world as well as his old neighbourhood on a television screen.

Arthur said that he felt completely content with life as long as he was able to get up in the morning and go for a walk while listening to the radio. This was supported by John who went for walks everyday – come rain or shine.

*“It happens automatically”,*

he explained, which implies that the activity is a habit. John and his partner had no social network and as both were on disability benefits they spent a lot of time together in their home. To John the daily walks gave rhythm and content to the day.

To some people, being physically active was a lifestyle. Several of the participants talked about being active throughout life and expressed that it was part of who they were. Alice, who only recently had moved into a nursing home, said:

*“I am crazy about mountains”.*

She explained how she had always loved walking in the mountains, and that they had a cabin where they used to spend lot of time. She also described that even though she preferred walking in the mountains, she often had to settle for a walk in the woods after she moved into a nursing home. It was obvious from the conversation that she considered herself as “sporty” and wanted to hold on to this identity.

One participant who had been active all his life explained how the objectives of the physical activities had changed as he aged.

*“It is no longer about the performance. When I ran and swam and so on, it was all about *performance*, but not now. Not on that level”.*

He held on to activities, but the focus had changed. He said further about exercising at a lower intensity level:

*“It’s got to do with getting older. It feels very natural, and I don’t feel bad about it”.*

He simply enjoyed the freedom of getting around on his bike or by foot – with or without his girlfriend.

Some participants talked about how they used exercise to benefit their health. In one instance, the person had taken up running after the dementia diagnosis because the doctor had advised him to, as it would be.

*“(…) good for his head”.*

He explained that he had been devastated when he received the Alzheimer diagnosis from his doctor, and quickly made a point of following the advice he gave him. Another participant emphasized that he did not need the doctor to tell him that it was good for him to be active – he knew that already! The effects they sought from being active were for some related to the dementia condition, and for others not. Mia explained that it was important to her to keep active because she was getting older and wanted to keep up her strength and stamina. That way they could keep travelling abroad and go to the cabin by the sea. Julie had experienced that her balance was deteriorating and had made a conscious effort to do exercises in the pool to reverse this loss of balance function. This was supported by several other participants who expressed that being active was a way to keep healthy – not only in regard to the dementia condition, but also in the context of getting older.

### To be active is challenging

This category describes how the participants encountered barriers in their endeavour to stay active or be active. Their reduced physical and cognitive function, the need of practical help and support and their perceived dependence on others are emphasized.

The participants talked about how their physical function affected their activity level. Some were restricted by reduced eyesight, pain, or simply lack of energy, while others were afraid of falling due to impaired balance. Julie explained that she used to enjoy being physically active, but after she had her hips replaced, she felt insecure about her balance. Pain was also reported as a reason for staying indoors and inactive. Emma spoke of backpain that not only stopped her from going outside, but also made it difficult to be social with her closest family. She said she rarely ventured outside the flat anymore. One participant experienced challenges with spatial orientation. Hugo said that he felt safe when he stuck to the same route and when he went for walks in his hometown. After getting lost and needing assistance to find his way back twice, he hesitated to move outside these boundaries.

Some of the participants were open about the fact that they more often than before felt indifferent and uninspired, and that they were less interested in activities and hobbies than earlier. They expressed that they had enjoyed specific physical activities when they were younger. When asked about this, Victor said:

*“Yes, I guess you could say that I miss being more physically active, but I just don’t have the *same* motivation as before”.*

Several were dependent on someone else to motivate them and remind them of their activities. Mia said that she was active thanks to her husband. Even though she enjoyed going for walks, she was bent towards settling on the coach with a book. She said that because her husband was very interested in exercising and keeping fit, she was motivated to join in. Some of the participants reported that they felt that their energy levels were lower than earlier, and that it took more effort than before to get out and be active. Karl was not sure why he no longer met up with the other neighbours for bowling. He expressed that he would have enjoyed keeping that activity up, but it was an exertion to get out of the house. The season and weather were also reported by the participants as a barrier to be active. Especially the darkness of early evening and wet weather during autumn and winter demanded additional motivation to initiate physical activities. Julie said that she was especially bored during the winter months as she tended to keep indoors.

The dependence on others was made apparent in different ways. Some participants who had lost their driving license found themselves completely reliant on partner or family members to take them where they wanted to go. Still in her seventies, Julie found it difficult to depend on her husband to drive her to activities. She talked about a neighbour who still drove while in her nineties, and Julie was not completely convinced that it was the correct decision to revoke her driving license. Julie expressed herself this way:

*“I feel that bothering my husband about driving me everywhere is taxing, even though he says it is ok. Earlier, I could just get in the car and go wherever I wanted”.*

The consequence of this was that the threshold for doing an activity was heightened, and thereby acted like a barrier for physical activity. Another way to be dependent on other people was highlighted by Kristian. He said that he was relying on help from a physiotherapist to be able to carry out the appropriate exercises at the right intensity. He emphasised how the physiotherapist demanded a lot from him, and in that way the exercises seemed to be more efficient than what he could manage by himself. Due to limited physiotherapy resources in the municipality, he had to endure periods between bouts of treatments without access to physiotherapy. This caused Kristian great distress as he felt he needed this service regularly.

The participants who lived in nursing homes also spoke of being dependent on others to keep active. Some expressed frustration about having to be accompanied when going for a walk outside because they felt it was unnecessary. Others would have liked to get out and moving more often but was denied this due to shortness of staff.

## Discussion

Traditionally, dementia research primarily has focused on cognitive concerns of the disease, but there is an emerging interest in providing insight into other aspects of living with a dementia condition. Huber et al. (2011) proposed a new definition of health as the ability to adapt and to self-manage when encountering social, physical and emotional challenges [39]. This shift in focus from symptoms and disability towards the capacity and potential of the person is in line with the desired direction of dementia care that emphasises on a balanced view of dementia to create a more dementia- and age- friendly societies [40]. Huber and colleagues proposed changing the emphasis towards the ability to adapt and self-manage in the face of social, physical, and emotional challenges and identified three aspects of health: physical, mental and social health. Involvement and inclusion in meaningful activities have been reported to influence all three dimensions of social health [41].

Taking pleasure in exercise and physical activity is key to staying motivated and engaged throughout life [42]. In the current study the participants said that being physically active was a source of enjoyment and positive experiences and was valued across the stages of dementia progression. This has also been found elsewhere [43].The participants described pleasant social situations, satisfying physical sensations after exertion and psychological wellbeing due to mastery. This concurs with findings from other studies [28, 44, 45]. Farina et al. (2020) demonstrated that people with dementia find that physical activity promote emotional wellbeing through being enjoyable and fulfilling the participants’ competitive nature [44]. One of their participants tracked their own progress at the gym and by doing so, he competed against himself. Participants in the current study described how they competed against themselves and others. Some were pleased about being fitter than their cognitively healthy peers, others were satisfied with still “measuring up”. The feeling of competency also promotes wellbeing and self-esteem. In people with dementia who suffer many losses and deteriorations, this may be of particular importance. Also, an association between self-esteem and feelings of hope has been demonstrated [46]. Hope is central to the adjustments process when trying to maintain a sense of normalcy, and in dementia hope is important to maintain well-being and quality of life [47]. Physical activity facilitates shifting focus from being a patient, and thus aiming attention at symptoms and limitations, to a physically capable individual [48]. The importance of the social aspect of exercise has also been demonstrated in other studies. Malthouse and Fox (2014) showed that the social interaction can be both a reason for joining an activity as well as a barrier for people with dementia. Because the focus is on the activity, there is less focus on conversation that can be a struggle for some people with dementia [29]. On the other hand, the lack of understanding by other people about dementia can lead to difficult situations and withdrawal from the activity.

The participants in the current study communicated that they wanted to maintain continuity in life, including their lifestyle behaviours. This has also been described by other researchers [49–51]. In agreement with this, the literature has repeatedly demonstrated that people with dementia express a desire to stay “normal” and stick to routines despite dementia condition, and that this can be considered a coping strategy [50, 52, 53]. The participants in the current study express a need to hold on to physical activity routines. To some, the routine is integrated into the daily routine to an extent that it “happens automatically”. This is important when people with dementia develop apathy which is often characterised by lack of initiative. Identities are closely tied to what we do [54] and when someone experiences cognitive decline, habitual daily routines may be a way to sustain a sense of self. Facilitating the continuity of lifestyle and physical activity choices for people with dementia can be important to maintain or restore a sense of personhood [55, 56]. The meaningfulness of activities is connected to the person’s psychological needs and these activities reinforce the sense of identity and belonging [57]. In the endeavour to support the self-image of people with dementia, it is imperative to emphasise on things that the person can still do – and likes to do, and at the same time, things that were important to the self-image in the past [58]. Phinney et al. (2007) found that it is important to identify the activities that people enjoyed before the onset of dementia in order to create opportunities for involvement in activities that have been “meaningful in the context of past experience and everyday life” [59]. This concurs with Atchley’s continuity theory. The continuity theory holds that older adults usually will maintain the same behaviours, activities and relationships as they did in their earlier years and that this is the way they adapt to changes during the life course [60]. Atchley further proclaimed that continuity of lifestyle, such as physical activity level, is very important when people develop psychologically and socially as they age.

In the current study, some of the participants found physical activity meaningful because they believed that exercise benefitted their health and condition. This has been confirmed in other studies as well [29, 55, 61]. A synthesis of qualitative studies suggested that engaging in, amongst other, physical activities, was meaningful to community-dwelling people with dementia because they believed that these activities would improve memory and restore their abilities [55]. This means that participants felt that they were in position to influence their health and their situation to a certain degree. This can be important to facilitate a need for a sense of control as well as achievement of life goals [62]. Many people with dementia consider physical exercise to be a way of maintaining physical function as they age, and several of our participants explained that this was particularly important now that they were getting older. Hence, information about the health benefits of exercise and physical activity is important to exercise engagement but the information needs to be individualised [42].

Van der Wardt (2019) found that barriers towards physical activity amongst people with dementia included health issues, low self-efficacy regarding physical activities and environmental factors which comprised the surroundings not being inspiring for walks and potential activities not being accessible [42]. The participants in the current study described that the greatest barriers to being physical active were declined function, lack of motivation or energy and dependence on others. Passive behaviours and apathy are frequently seen in persons with dementia [63] and this reinforces the dependence on others to take initiative. Apathy in nursing home has been found to be associated with a more restricted life space [64]. At the same time, as described in our results, people with dementia often lose their driving privilege during the course of disease which adds to the dependence on others for transport and practical assistance. Both van Alphen et al. (2016) and Farina (2020) found declined physical function to be a barrier for people with dementia to be physical active [44, 65]. This was also reported in the current study. This demonstrates the importance of developing physical activity services that meet the need of people with different level of physical function. One of the participants who lived in a nursing home during her interview said that her access to walking outdoors were reliant on the nursing staff having the opportunity to go with her. The limited life space in nursing home residents has been reported elsewhere [66]. Regretfully, only two of the participants were nursing home residents, so the material cannot advise us further on this subject.

The results from the current study give emphasis to the role of the carer in motivating and facilitating physical activity in the person with dementia. This is supported by van Alphen et al. (2016) and Farina (2020) [44, 65]. Our results showed that it was imperative to some of the participants that family carers encouraged and facilitated physical activities. Without this facilitation from the informal carers, some activities might not be possible to carry out [44]. The World Health Organization established social support as a crucial element in its Global Action Plan as a public health response to dementia in 2017 [67]. A systematic review demonstrated that social support for physical activity was associated with level of physical activity in older adults, especially social support from family members [68]. The significance of the carer in motivating and supporting physical activity in people with dementia has been raised by several researchers [53, 65]. Social support can be subdivided into two main components: structural support and perceived support [69]. Perceived social support is most reliably associated with psychosocial and cognitive function and it has been found to be less in individuals with mild dementia than in persons with advanced cognitive deficits [70]. The authors suggested that this might reflect specific needs of individuals with a mild dementia condition who may feel threatened by the onset of changes. Social interaction with others, such as the informal carer, can also serve as a great motivator to take part in exercise class [71]. Hence, caregivers’ attitudes toward exercise influences the participation in regular physical activity and exercise [61]. Informal carers often worry about that the person with dementia becomes passive and informal carers describe a lack of meaningful and stimulating activities in day care for people with dementia [72].

### Strength and limitations

The twenty-seven participants in this study are heterogenous regarding gender, age, living situation, interests, physical function, and severity of dementia disease. This means that a range of different voices have been raised about physical activity. Type of dementia disease was not recorded, and we did not assess cognition. Most interviews were carried out in the persons’ home and the atmosphere was mostly comfortable and positive. In the interviews where next of kind were present due to severity of dementia disease and/ or hearing difficulties (*n* = 9), a few participants were cut short, or answered for, by eager family members even though the interviewer tried to minimise this. The restricted interview experience of the first author is a limitation as practice is important to develop interview skills, however two of the co-authors had extensive interview experience with this patient group. The active participation of three authors in the data analysis process is a strength.

## Conclusion

An overall synthesis of the results revealed a comprehensive latent theme: Despite the challenges of living with dementia, physical activity is a source of identity confirmation, mastery and enjoyment. The conversations with the participants demonstrated that many of them had positive experiences with physical activities, and the activities were considered important and meaningful. Facilitating participation in meaningful physical activities is a critical aspect of supporting people with dementia. To do this it is imperative to know what the persons find meaningful. Communicating with the person with dementia as well as family and friends is key to access this information. Barriers to being physically active were also reported on by the participants. To ensure equal opportunities to participation in physical activities, and to healthy ageing, it is important that both formal and informal carers are aware of experienced barriers so that appropriate measures can be taken.

## Supplementary Information

**Additional file 1.**

## Data Availability

The datasets generated and analysed during the current study are not publicly available due the maintenance of confidentiality of our participants and declarations within the written information which participants had agreed on.
