# Supplementary material for: Fun and a meaningful routine: the experience of physical activity in people with dementia
Source: BMC Geriatr. 2022 Jun 10;22:500. doi: 10.1186/s12877-022-03149-6 (PMC9188090; doi:10.1186/s12877-022-03149-6)
Supplement: Supplementary file 1 — Additional file 1. [file 12877_2022_3149_MOESM1_ESM.pdf]

Interview guide

Part 1: Introduction and background

<u>Information about the project</u>

Consent form

Data collection – how, where, when. Audio recording and storage of data.

What will the data be used for?

Anonymity, professional confidentiality

<u>Background information</u>

Age, education, work, marital status, services received

Part 2: Information and emotional needs

<u>Needs during diagnostic process and need for information</u>
Your experience with the diagnostic process?
Before: What changes did you notice, or the carer?
Being informed about diagnosis. How did you experience this? What information did you get? What information was useful? Sufficient information?

Your situation now? Thoughts about the future?
Was anything particularly useful or did you miss something in this process?

What kind of help have you received – prior to diagnosis and now? What do you think you will need in the future?
What is your experience with receiving help and asking for help?
Can you influence the services that you receive and how they are carried out?
Which professional groups have you been in contact with since the diagnosis?

Key words: Safety, social needs, comfort, respect and attachment, medicine, loss, mastery, communication, self-esteem.

Need for activity/ entertainment/ relaxation/ social life

What is important to you during daily living?

What do you like to do? What do you appreciate?

Is it different from before?

Relations

Who are important in your life? Any changes?

What do you need to live a meaningful life? What is a “good life” to you?

Specific activities that you would like to keep up? How?

Are you physically active? Were you active earlier?

Key words: work, day care, isolation, hobby, projects, sports, arts, music, social life, finance, autonomy, religion/ faith, welfare technology

---

Part 3: User involvement

In what way do you wish to participate in decisions that affects you?

Can someone make decisions for you? Who? Circumstances?

Key words: Treatment, living arrangements, facilitation, services, advance care planning, legal issues

---

Part 4: Summary

Thank you for your participation and your willingness to share your experience. A lot of what you have shared with me have been interesting and important.

Summing up…

Did I get it right? Did you mean…?

Is there anything you would like to add? Things we have not talked about?

Thank you!
